# Primary Healthcare Practitioners’ Knowledge, Attitude, and Practice Toward Systemic Lupus Erythematosus in the Qassim Region, Saudi Arabia

**DOI:** 10.7759/cureus.30297

**Published:** 2022-10-14

**Authors:** Mousa N Alrashdi, Sami M Alrasheedi, Ahmad Alkhdairi, Faisal N Alburaq, Almutairi E Muteb, Abdulaziz S Alshamikh, Khalid Almutairi, Almutairi N Ammar, Almutairi L Saleh, Alfurayhidi W Nawaf

**Affiliations:** 1 Department of Medicine, Unaizah Collage of Medicine and Medical Sciences, Qassim University, Unaizah, SAU; 2 Internal Medicine, Qassim University, Unaizah, SAU; 3 College of Medicine, Qassim University, Unaizah, SAU

**Keywords:** saudi arabia, qassim, systemic lupus erythematosus, sle, attitude, knowledge, primary health care

## Abstract

Objective

The study aims to evaluate the primary healthcare practitioner’s (PHCP) knowledge, attitude, and practice toward systemic lupus erythematosus (SLE) and to explore the difficulties of early referral to specialized clinics.

Method

This is a cross-sectional study conducted between February and March 2022 targeting the PHCP among the primary healthcare centers in Qassim, Saudi Arabia. The study was conducted among 203 participants who enrolled via a cluster random sampling technique depending on the survey. Qualitative categorical variables are expressed as frequencies and percentages, while continuous variables are reported as means and standard deviations (SD). The one-way analysis of variance (ANOVA) test and the independent T-test have been used to explore the relationship between participants' knowledge scores and their socio-demographic characteristics. A P-value ≤0.05 was considered statistically significant.

Result

The study found that only 4.4% of participating PHCPs had good knowledge related to SLE, while 45.8% of them had fair knowledge, and nearly half of them (49.8%) had poor knowledge. It was noted that GPs who specialized in family medicine or saw more than 40 patients each week had comparably better knowledge than others, which was statistically significant (p<0.05). The difficulty in diagnosing SLE patients was mentioned by 48.3% of the PHCPs. Family medicine consultants (p<0.001), those who worked in a tertiary care facility for more than six months (p<0.001) and those who worked in a rheumatology department (p<0.05), reported considerably better levels of comfort when treating SLE patients.

Conclusion

This study found that included participants' understanding of SLE, its diagnosis, and management was inadequate. Coordination between rheumatologists and experts from many disciplines at the primary, secondary, and tertiary levels of care is critical for overcoming ambiguities and obstacles in the diagnosis and therapy of SLE patients.

## Introduction

Systemic lupus erythematosus (SLE) is an autoimmune multisystem disease that affects the majority of the body organs, producing different clinical manifestations [1]. The estimated prevalence of SLE in the Qassim region of Saudi Arabia was found to be 19.28 per 100,000 people [2]. Patients may exhibit a wide range of systemic manifestations or general symptoms, such as fever, weight changes, and fatigue, as well as more specific cutaneous manifestations such as rash, photosensitivity, discoid rash, and alopecia [3]. Moreover, patients may also present with musculoskeletal symptoms like arthralgia, myalgia, or inflammatory arthritis; hematological involvement (leukopenia, anemia, or thrombocytopenia); renal involvement (lupus nephritis); pulmonary involvement (pleurisy); and central nervous system involvement (headache, psychiatric, and other neurological symptoms) [3,4]. The European League Against Rheumatism (EULAR) and the American College of Rheumatology have established current classification criteria for SLE diagnosis (ACR). The presence of positive anti-nuclear antibodies (ANA) at least once, as well as an additional seven clinical and three immunologic features expressed as scores ranging from 2 to 10, is necessary for the diagnosis of SLE [5]. EULAR 2019 updates for SLE treatment recommendations have been developed to improve long-term patient outcomes; they mainly depend on disease activity and organ involvement, which include hydroxychloroquine, glucocorticoids, immunosuppressive, and biological agents [6]. It is crucial to diagnose SLE as early as possible because early detection of SLE patients leads to better clinical results and is also cost-effective as it lowers expenditures [7]. Despite increased awareness of SLE, the average period from symptom onset to diagnosis remains about two years [8]. Delays in receiving a definite diagnosis and commencing immunosuppressive therapy have been associated with poor outcomes in SLE patients with significant organ involvement [9,10]. Additionally, failure to attain minimum disease activity during the first six months after diagnosis has also been associated with early damage development [11,12]. Finally, appropriate therapy can enhance all subscales of quality of life in individuals with early illness over a two-year period in patients with early disease [13]. Nonetheless, the role of primary healthcare practitioners (PHCPs) in SLE is increasing since there are still important unmet health demands, such as community-level diagnosis delays and the high burden of therapy- and disease-related comorbidities. The goal of an integrated lupus care paradigm, particularly between PHCPs and specialty facilities, should undoubtedly improve patient consultations and outcomes [14]. It has been noted that general practitioners (GPs) correctly diagnosed only 11% of SLE cases presented in clinical scenarios, far fewer than rheumatologists [15]. Furthermore, SLE awareness research in Cotonou, Benin demonstrated that the majority of GPs had minimal comprehension and awareness of the clinical and diagnostic criteria for SLE, while another study in China discovered that more than half of them were unaware of SLE [16,17]. Despite several studies on the Saudi general population's comprehension of SLE, no studies on PHCPs' understanding of SLE have been conducted in the Kingdom of Saudi Arabia (KSA). As a result, the purpose of this study is to assess our present PHCP's knowledge and attitude toward diagnosing and managing SLE. Additionally, we look at potential sources of deficiency and difficulties in these areas.

## Materials and methods

Setting and participants

This is a cross-sectional study based on a closed, fixed-response interview survey. Interviewers used an electronic-based questionnaire built using GoogleForms™ and targeting PHCPs in primary healthcare centers in the Qassim region, KSA. We targeted 203 participants as a sample size that was calculated for a descriptive study by using EpiInfo™ (Centers for Disease Control and Prevention (CDC), Atlanta, Georgia). In order to collect the targeted sample, the questionnaire was emailed to 263 physicians. The technique was a cluster random sampling technique where lists of names and emails of PHCPs who are currently working in the Qassim region were obtained from the Qassim Health Cluster. Then the physicians were clustered into groups according to qualification level and finally coded as numbers, which are used to collect data from each cluster randomly.

Data collection methods

A newly developed questionnaire was constructed from three main domains: the first domain includes demographic data and current practice; the second domain includes 28 questions that assess the knowledge about SLE subdivided into three sections, which include clinical presentation and organ involvement, diagnostic competency, and lastly, management plan. The third domain discussed the difficulty faced by PHCPs in managing and refereeing patients to specialized centers and their suggestions for improvements.

The draft of our initial questionnaire was made in the English language, which had 31 items excluding sociodemographic characteristics. Three experts, including a biostatistician, assessed the content and face validity by focused group discussion. This group had several revisions of the questionnaire to make it relevant and put it through many meetings. The items that did not agree to have face validity and/or content validity were eliminated. This initial questionnaire was subjected to pilot testing on 20 participants for validity and reliability. An exploratory factor analysis was performed to check the construct validity of the questionnaire. Items with a correlation coefficient >0.7 were removed. For reliability analysis, the items with "uncertain" answers were marked as negative answers. Internal consistency was measured using Cronbach’s alpha and test-retest reliabilities between the initial and final questionnaires for each item were calculated using kappa statistics. Cronbach’s alpha, which was calculated along with one-sided 95% CL. A Cronbach's α value >0.7 was considered for our questionnaire to be internally consistent.

Data analysis plan

The Statistical Package for Social Sciences (SPSS) software version 26 (IBM Corp., Armonk, NY) is used to code, enter, and analyze data. Quantitative categorical variables are expressed as frequencies and percentages, while continuous variables are reported as means and standard deviations (SD). There were 28 items that measured knowledge related to SLE, and the total knowledge level of the participants was calculated using scores based on the correct responses. For items that had only one option as the correct answer, participants were allotted a score of 1 for a correct response and a score of 0 for a wrong response. For items that had multiple options as correct answers, score 1 was divided by the number of correct options. For example, if there are four correct answers for an item, the participants get a full score of one if they choose all four correct options; they get a 0.75 score if they choose three correct options. The knowledge level was determined by converting the total scores into percentages and categorizing them as good knowledge (>75%), fair knowledge (60-75%), and poor knowledge (60%). Demographic factors included age (<30, 31-35, 36-45, 46-55, and > 55), gender (male, female), qualification level (general physician, master in family medicine, family medicine specialist, family medicine consultant, and other), experience (0-2, 3-5, 6-10, >10 years), patients seen per week (0-20, 21-40, >40), SLE patients seen per year (0, 1-10, 11-20, >20), working at TCC for more than 20 years (Yes, No), and SLE patients seen per year (Yes, No). The one-way analysis of variance (ANOVA) test, the independent T-test, and the chi test were used to explore differences between participants' knowledge scores according to their socio-demographic characteristics. A P-value of 0.05 or less was considered statistically significant.

## Results

We received 203 responses to the interview survey (a response rate of 77.18%). The participants' sociodemographic characteristics showed that 31.5% belonged to the age group of 35-45 years, 58.1% were males, 68.5% were GPs, and 38.9% had experience of more than ten years. The practice-related characteristics showed that 70.4% of the participants saw >40 patients per week; 55.2% reported that they saw 1-10 SLE patients in a year; 43.3% reported that they worked in a tertiary care center (TCC) for more than six months; and 11.8% worked in a rheumatology department (Table 1).

**Table 1:** Sociodemographic and practice-related characteristics ^1^Systemic lupus erythematosus. ^2^Tertiary care center.

		N	%
Age (years)	<30	51	25.1
	31–35	22	10.8
	36–45	64	31.5
	46–55	50	24.6
	>55	16	7.9
Gender	Female	85	41.9
	Male	118	58.1
Qualification level	General physician	139	68.5
	Master in family medicine	23	11.3
	Family medicine specialist	23	11.3
	Family medicine consultant	14	6.9
	Others	4	2.0
Experience (years)	0–2	41	20.2
	3–5	34	16.7
	6–10	49	24.1
	>10	79	38.9
Patients seen per week	0–20	24	11.8
	21–40	36	17.7
	>40	143	70.4
SLE^1^ patients seen per year	0	77	37.9
	1–10	112	55.2
	11–20	10	4.9
	>20	4	2.0
Worked at TCC^2^ for more than 6 months	No	115	56.7
	Yes	88	43.3
Worked in a rheumatology department	No	179	88.2
	Yes	24	11.8

The assessment of knowledge related to SLE showed that 95.1% agreed that SLE is an autoimmune disease, and 83.3% responded that it affects females predominantly. The correct responses related to other knowledge items regarding SLE, its diagnosis, and management are given in Table 2.

**Table 2:** Participants responses for knowledge-based items related to systemic lupus erythematosus (N=203) ^1^Systemic lupus erythematosus. ^2^European League Against Rheumatism and the American College of Rheumatology. ^3^Primary health care. ^4^Corticosteroids. ^5^Hydroxychloroquine. *Wrong answers.

	Correct responses n (%)
SLE^1^ is an autoimmune disease	193 (95.1)
SLE disease predominantly affect female gender	169 (83.3)
SLE disease commonly affecting patient between the age of 20 and 50	170 (83.7)
SLE can be induced with exposure to some of medications	156 (76.8)
Constitutional symptoms include (fatigue, fever, and weight loss) can be presented in SLE patient	181 (89.2)
SLE patients can present with renal manifestations like proteinuria and glomerulonephritis	191 (94.1)
SLE patients can present with serositis including pleural effusion, pericardial effusion, and pericarditis	163 (80.3)
SLE patients can present with musculoskeletal symptoms include (weakness, myalgia, arthralgia, arthritis, and morning stiffness)	188 (92.6)
SLE patients can present with cutaneous skin manifestation like alopecia, oral ulcers, and skin rash	190 (93.6)
SLE patients can present with hematological manifestations like (anemia and leukopenia)	163 (80.3)
Almost all patients of SLE present with neuropsychological symptoms like (delirium, seizures, and psychosis)*	102 (50.2)
SLE disease is not associated with antiphospholipid syndrome*	62 (30.5)
Know a lupus specific skin disease	78 (38.4)
Know the classification criteria for SLE	32 (15.8)
2019 EULAR/ACR^2^ criteria that have high sensitivity for SLE classification	41 (20.2)
2019 EULAR/ACR criteria that have high specificity for SLE classification	45 (22.2)
Clinical scenario that is highly possible as SLE case	122 (60.1)
Screening test is recommended to use in suspected SLE case	148 (72.9)
Tests specific for SLE disease	23 (11.3)
Leukopenia, anemia, and thrombocytopenia are used as a marker of disease activity in SLE patients	117 (57.6)
Antinuclear antibodies is used as a marker of disease activity in SLE patients*	31 (15.3)
Anti-double strand DNA and complements level are used as a marker of disease activity in SLE patients	132 (65)
Raised creatinine level and presence of active sediments or proteins in urine sample is used as a marker of renal involvement in SLE patients	153 (75.4)
Treating patients with SLE in multidisciplinary clinics will lead to better outcomes than treating them by PHC^3^ provider alone	171 (84.2)
It’s recommended to start CS^4^ therapy immediately in newly diagnosed SLE patients regardless their disease activity*	46 (22.7)
Anti-malarial medication like HCQ^5^ is recommended to all SLE patients due to mortality and morbidity benefit	143 (70.4)
Medications that is considered safe and should be continue during pregnancy in SLE patients	36 (17.7)
It is safe for SLE patients with quiescent disease to receive Covid-19 vaccine	126 (62.1)

**Figure 1:**
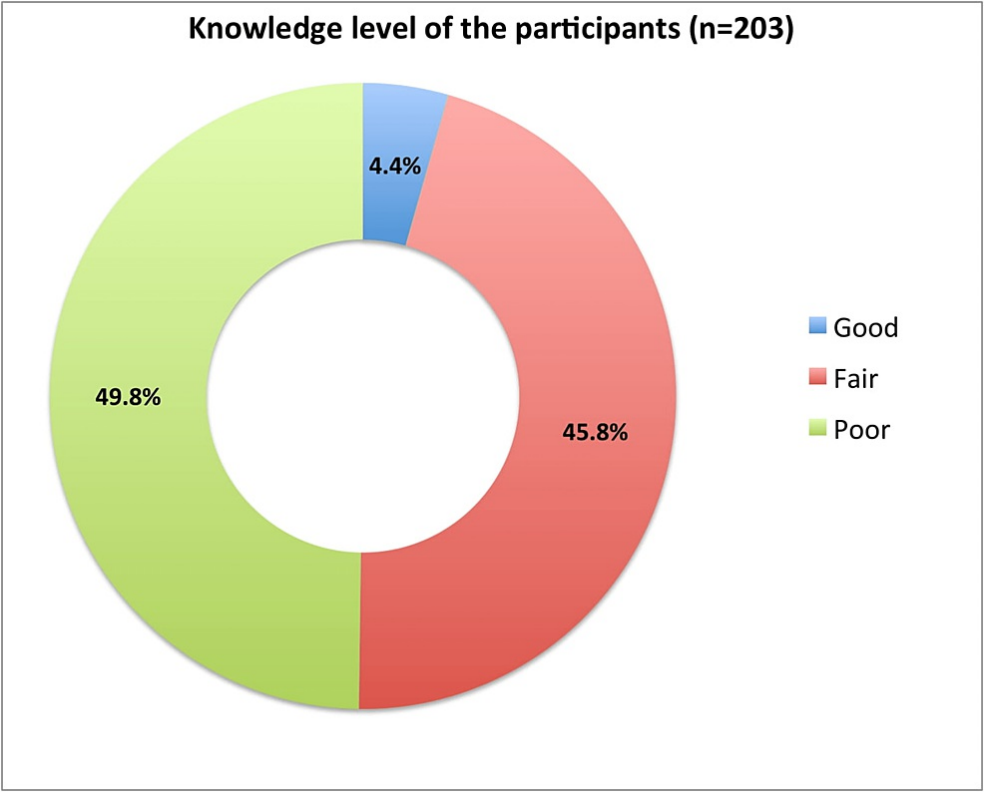
The knowledge level of the participants toward systemic lupus erythematosus, good knowledge (>75%), fair knowledge (60-75%), and poor knowledge (60% or lower).

The analysis showed that the mean knowledge score of the participants was 15.95 ± 4.12 (minimum: 0 and maximum: 25.25). Our study found that only 4.4% had good knowledge related to SLE, and nearly half of the participants (49.8%) had poor knowledge (Figure 1).

The relationship between the knowledge level and various sociodemographic and practice-related characteristics is given in Table 3. It was found that participants who had the qualification level of family medicine specialist and family medicine consultant demonstrated comparatively higher knowledge than others, but without a significant difference (P = 0.068). The participants who had seen more than 40 patients per week showed better knowledge levels than those who had seen a lesser number of patients, however, without a significant difference (p = 0.448). The participants' age, gender, and other practice-related characteristics did not show any statistically significant differences in knowledge levels (Table 3).

**Table 3:** Knowledge levels and their relationship with sociodemographic and practice-related characteristics ^1^Systemic lupus erythematosus. ^2^Tertiary care center. ^α^Bonferroni adjusted p-value (ANOVA test). ^β^P-value as calculated by Chi-test.

		Knowledge						P-value
		Good		Fair		Poor		
		Count	Row N %	Count	Row N %	Count	Row N %	
Age (years)	<30	4	7.8%	16	31.4%	31	60.8%	0.208^α^
	31–35	1	4.5%	9	40.9%	12	54.5%	
	36–45	2	3.1%	31	48.4%	31	48.4%	
	46–55	2	4.0%	31	62.0%	17	34.0%	
	>55	0	0.0%	6	37.5%	10	62.5%	
Gender	Female	5	5.9%	41	48.2%	39	45.9%	0.517 (1.32)^β^
	Male	4	3.4%	52	44.1%	62	52.5%	
Qualification level	General physician	7	5.0%	57	41.0%	75	54.0%	0.068^α^
	Master in family medicine	0	0.0%	11	47.8%	12	52.2%	
	Family medicine specialist	2	8.7%	11	47.8%	10	43.5%	
	Family medicine consultant	0	0.0%	13	92.9%	1	7.1%	
	Others	0	0.0%	1	25.0%	3	75.0%	
Experience (years)	0–2	3	7.3%	13	31.7%	25	61.0%	0.116^α^
	3–5	2	5.9%	12	35.3%	20	58.8%	
	6–10	1	2.0%	21	42.9%	27	55.1%	
	>10	3	3.8%	47	59.5%	29	36.7%	
Patients seen per week	0–20	0	0.0%	15	62.5%	9	37.5%	0.448^α^
	21–40	4	11.1%	8	22.2%	24	66.7%	
	>40	5	3.5%	70	49.0%	68	47.6%	
SLE^1^ patients seen per year	0	1	1.3%	32	41.6%	44	57.1%	0.201^α^
	1–10	8	7.1%	54	48.2%	50	44.6%	
	11–20	0	0.0%	5	50.0%	5	50.0%	
	>20	0	0.0%	2	50.0%	2	50.0%	
Worked at TCC^2^ for more than 6 months	No	7	6.1%	45	39.1%	63	54.8%	0.062 (5.57)^β^
	Yes	2	2.3%	48	54.5%	38	43.2%	
Worked in a rheumatology department	No	8	4.5%	78	43.6%	93	52.0%	0.209 (3.13)^β^
	Yes	1	4.2%	15	62.5%	8	33.3%	

It was reported by 48.3% of the participants that they addressed the difficulty in diagnosing SLE patients, whereas those not specialized in family medicine significantly reported greater difficulty compared to others (p<0.05). It was also found that participants, those who had experience (less than five years) and those who had not worked in the rheumatology department before, had difficulty in diagnosing SLE (p<0.05). It was reported by 20.2% that they had difficulty in referring SLE patients to specialized centers, and this was significantly more seen in GPs than others (p<0.05) (Table 4).

**Table 4:** Participants responses regarding systemic lupus erythematosus diagnosis and referrals difficulties ^1^Systemic lupus erythematosus. ^2^Tertiary care center.

		N (%)	Qualification level	Experience	Patients seen	SLE^1^ patients seen	Worked in TCC^2^ for >6 months	Worked in rheumatology department
			P-values					
Difficulty in diagnosing SLE patients	No	105 (51.7)	0.013	0.039	0.073	0.082	0.204	0.015
	Yes	98 (48.3)						
Difficulty in referring SLE patients to specialized centers	No	162 (79.8)	0.014	0.067	0.733	0.132	0.255	0.934
	Yes	41 (20.2)						

The degree of comfort in treating SLE patients alone was measured using a scale of 0 to 5, where 0 showed "not comfortable" and 5 was "very comfortable." The mean comfort score was found to be 2.14 ± 1.5. When we compared these scores between different age groups, it was found that participants aged 46-55 years had given significantly higher scores than others (p<0.05). The comfortableness scores were significantly higher among family medicine consultants (p<0.001), those who worked in a TCC for more than six months (p<0.001), and those who worked in a rheumatology department (p<0.05) (Table 5).

**Table 5:** Comparison of comfortableness in treating SLE patients alone ^1^Systemic lupus erythematosus. ^2^Tertiary care center. *Significant at p-value <0.05. ^α^ANOVA test Bonferroni corrected P-value. ^β^t-test P-value.

		No.	How comfortable are you in treating SLE patients alone?		
			Mean	Standard deviation	P-value
Age (years)	<30	51	1.9	1.5	0.008*^α^
	31–35	22	2.2	2.0	
	36–45	64	1.9	1.3	
	46–55	50	2.8	1.4	
	>55	16	1.9	1.0	
Gender	Female	85	2.0	1.5	0.242^β^
	Male	118	2.2	1.4	
Qualification level	General physician	139	2.1	1.5	0.000*^α^
	Master in family medicine	23	1.5	1.0	
	Family medicine specialist	23	1.8	1.1	
	Family medicine consultant	14	4.1	.8	
	Others	4	1.8	1.5	
Experience (years)	0–2	41	2.0	1.7	0.853^α^
	3–5	34	2.1	1.3	
	6–10	49	2.1	1.5	
	>10	79	2.3	1.4	
Patients seen per week	0–20	24	2.0	1.3	0.732^α^
	21–40	36	2.0	1.3	
	>40	143	2.2	1.5	
SLE^1^ patients seen per year	0	77	2.0	1.5	0.840^α^
	1–10	112	2.2	1.5	
	11–20	10	2.1	1.0	
	>20	4	2.3	1.7	
Worked at TCC^2^ for more than 6 months	No	115	1.7	1.4	0.001*^β^
	Yes	88	2.7	1.4	
Worked in a rheumatology department	No	179	2.1	1.5	0.014*^β^
	Yes	24	2.8	1.2	

## Discussion

SLE's clinical profile is typically challenging due to the disease's unpredictable nature, which affects many organs with different levels of severity and is worsened by the consolidation of organ damage and comorbidities [18]. The findings of our study showed that the knowledge related to SLE, its diagnosis, and management was not satisfactory among the participants, as only 4.4% of those sampled had demonstrated good knowledge. According to Art et al., many GPs are concerned about their lack of expertise and experience in dealing with SLE, despite technological advancements in diagnosis, and hence tend to overestimate the disease's possible impact on patients [19]. A recent study done in Cotonou, Benin reported similar findings as our results where the majority of the GPs had limited knowledge about SLE [16]. In another study, cutaneous SLE was reported to be one of the most difficult diagnoses for GPs compared to other skin disorders [20]. In our study, more than half of the PHCPs demonstrated poor knowledge related to SLE related-skin manifestations. The findings of our study showed that GPs had greater difficulty in diagnosing and referring SLE compared to specialists and consultants. We attribute this finding to a variety of factors, including GPs' lack of clinical training following their internship, as opposed to specialists and consultants, who typically complete their training in family medicine programs over four years, with at least rheumatology or internal medicine rotations. A lack of medical educational initiatives, such as lectures and conferences regarding rheumatological illnesses, particularly SLE aimed toward PHCPs, might potentially play a role. Traditional manual referral methods, inadequate tracking systems, and a lack of efficient coordination among healthcare practitioners may all contribute to the difficulty in sending patients with SLE to specialist treatment facilities. We believe that enhancing PHCPs' access to specialist consultation services in tertiary institutions and strengthening the referral system through the use of unified national medical records technology would help to overcome these difficulties. Secondary and tertiary centers with expertise in SLE have traditionally been used to diagnose and manage the disease to ensure timely medication initiation, early detection and control of flare-ups, and improvement of medical care throughout the disease trajectory [21]. The selection of substantially homogenous groups of patients for participation in research studies and trials requires classification criteria, and in our study, only 15.8% of the participants agreed that they knew some classification criteria for SLE. Many SLE classification criteria have been used, such as the 1982 revised ACR criteria and its revision in 1997, the 2012 SLICC, and the latest 2019-EULAR/ACR criteria, which are the most widely followed criteria [5,22-24]. The 2019-EULAR/ACR criterion has shown high sensitivity and specificity for SLE classification compared to the ACR-1997 and SLICC-2012 criteria [5,25]. Our analysis showed that only 20.2% and 22.2% correctly identified the same sensitivity and specificity of the 2019-EULAR/ACR criterion, respectively. The immunological hallmark of SLE is ANA positivity, which is primarily used for screening purposes, and the pattern of autoantibodies expressed by individuals with this prototypic autoimmune disease is highly distinctive [26,27]. In our survey, 15.3% of participants believed that ANA was employed as a measure of disease activity rather than a screening test in SLE patients. In primary health care, the diagnosis of SLE is often challenging because of many non-specific symptoms such as fatigue, rash, joint pain, etc. The biomarkers are often negative or normal in the early course of the disease [28]. Approximately 90% of SLE patients experience constitutional symptoms such as fatigue, weight loss, and fever without a focal infection. Other common manifestations seen in most patients include arthralgia and myalgia [29]. Malar rash and photosensitivity are reported to be present in 31% and 23%, respectively [28]. Other uncommon manifestations include the Raynaud phenomenon, pleuritic chest pain, and mouth sores [30]. The analysis showed that participants who had more experience and practitioners who had a history of working in the rheumatology department and TCC for more than six months demonstrated more comfort in treating SLE patients than others. It is reported that there is a paucity of specific understanding of SLE by family physicians and a need for more cohesive health care [31]. There is a scarcity of studies exploring how GPs and PHCPs refer to and write prescriptions for SLE management. Patients with SLE may have diagnostic and treatment challenges due to regional variations in the availability and accessibility of rheumatologists, as well as an overall shortage of rheumatologists [32,33]. To optimize chronic care and preventive health services for these patients, well-coordinated multidisciplinary healthcare teams comprised of subspecialists and family doctors are essential. ACR recommends that PHCPs and GPs understand the symptoms of SLE to aid in early diagnosis, treat and monitor patients with mild disease, acknowledge warning signs to accurately refer to a rheumatologist, and assist in evaluating clinical symptoms and therapeutic interventions in patients with moderate to severe disease [34].

Study limitations

Our study poses some limitations. First, the study only evaluated the level of knowledge without assessing its influence on the attitude of participants' practice, which could reduce the reliability of the findings. However, to the best of our knowledge, this was the first study done in KSA to assess the knowledge of PHCPs and comfort regarding SLE diagnosis, its management, and referrals. Second, we were unable to verify if the respondents were demographically representative of the total sample. We encourage future research on this topic to include practitioners from all provinces in KSA as well as investigate areas of weakness, such as the effectiveness of continuous medical education in addressing this serious disease, with the goal of improving their knowledge, which may positively reflect on patient outcome.

## Conclusions

This study showed that knowledge regarding SLE, its diagnosis, and management was not that satisfactory among the PHCPs. Family medicine consultants showed significantly higher levels of comfort in treating patients with SLE compared to GPs and other practitioners. To overcome ambiguities and challenges in the diagnosis and management of SLE patients, coordination between rheumatologists and specialists from different disciplines at primary, secondary, and tertiary levels of care is of paramount importance. The role of PHCPs is crucial in diagnosing both mild and severe SLE manifestations and guiding these patients, minimizing the community's disease burden.
